# Editorial: The Role of Microorganisms in Multiple Myeloma

**DOI:** 10.3389/fimmu.2022.960829

**Published:** 2022-06-30

**Authors:** Maria Linares, Sylvie Hermouet

**Affiliations:** ^1^ Pharmacy School, Universidad Complutense de Madrid, Madrid, Spain; ^2^ Department of Translational Haematology, Hospital 12 de Octubre de Madrid, Madrid, Spain; ^3^ Laboratory of Haematological Tumours, Centro Nacional de Investigaciones Oncológicas (CNIO), Madrid, Spain; ^4^ Nantes Université, INSERM, Immunology and New Concepts in ImmunoTherapy, INCIT, UMR 1302, Nantes, France; ^5^ Laboratoire d’Hématologie, Centre Hospitalier Universitaire de Nantes (CHU Nantes), Nantes, France

**Keywords:** myeloma, microorganisms, infection, autoimmunity, microbiota, MGUS, chronic antigen stimulation

Multiple myeloma (MM) is a bone marrow malignancy characterized by clonal, abnormal plasma cells that secrete large amounts of a single (monoclonal) immunoglobulin (Ig) (1). Despite great advances in knowledge and treatment, MM remains an incurable disease. MM is always preceded by a benign stage, called monoclonal gammopathy of undetermined significance (MGUS) (2). Recent studies suggested association of MGUS and MM with autoimmunity and with various microorganisms, including hepatitis C virus (HCV), human immunodeficiency virus (HIV), Epstein Barr virus (EBV), and other infectious pathogens (3–10). Indeed, chronic stimulation by self- or infectious antigens is recognized as an initial pathogenic event leading to chronic inflammation, cell proliferation, acquisition of genetic alterations, and cancer. This pathogenic model is proven in B-cell malignancies such as chronic lymphocytic leukaemia or lymphomas (11–13). Chronic antigen stimulation of B-cells triggers signaling pathways, increases proliferation and reduces apoptosis. Moreover, the complex and interactive balance between non-pathogenic microbes and the human immune system creates a steady state of coexistence, and disturbance of this balance may lead to illness. Direct links between the gut microbiota and MM have been suggested but its implication in the development of MGUS and MM remains poorly understood (14–16). In this collection of 1 original article and 3 reviews by 22 authors, the first section presents recent knowledge on the role of infectious pathogens in the initiation of MM, and the consequences for the therapy of this malignancy. The second section is devoted to the role played by the microbiota in MGUS and MM.

## Role of Infectious Pathogens

Many studies reported an increased prevalence of MGUS and MM in individuals with a prior history of auto-immunity or infections, supporting a role for chronic antigen stimulation in the pathogenesis of MGUS and MM (17–19). Sigurbergsdóttir et al. detail then summarize the reported links between autoimmune diseases, chronic inflammatory conditions, infections, and increased risk of MGUS and MM. The studies suggest that chronic stimulation by infectious antigens might trigger IgH-translocation in clonal plasma cells. The authors also carefully discuss the bias and methodological limitations of some studies, and the interest for prognosis and treatment of MGUS and MM of conducting further research on the association of auto-immune disease, infection and MGUS. Notably, a lower risk of progression to MM was found in MGUS patients with preceding autoimmune disease, compared with other patients (19). For MGUS and MM cases linked to HIV or HCV infection (20–22), the potential consequences for the therapy of MGUS (not treated presently) and MM are major: in pathogen-driven malignancies, when pathogen and antigen reduction or suppression is achieved, typically the associated malignancy is also reduced, or cured, including in MM (23–25). Rodríguez-García et al. demonstrate that this therapeutic approach is valid for HCV-linked monoclonal gammopathies, in MGUS and also in MM. They report that the monoclonal Ig from 6/9 HCV-positive patients reacted against HCV, and 4/6 patients who received antiviral treatment had a better evolution than untreated patients. Moreover, following antiviral treatment, one MM patient in third relapse achieved long-term complete remission. Thus, elimination of HCV led to the disappearance of antigen stimulation, facilitating the control of clonal plasma cells. This opens new possibilities of treatment for MGUS and MM linked to other treatable pathogens, such as HBV or *Helicobacter pylori* (26–28).

## Role of the Microbiota

Presently, the infection which drives MGUS or MM can be identified in 60% MGUS patients and 33% of MM patients (9, 10). As disease progression has been associated with dysbiosis in mice (14), Jasiński et al. suggest that it may be driven by gut microbiota. They hypothesize that pathogenic gut species could be responsible for chronic antigenic stimulation in subsets of MGUS and MM, mediated by presenting dendritic cells. Together with Brevi et al., the authors highlight the role of T cell differentiation within the gut towards Th17 cells, pathogenic in MM. This mechanism has been replicated in laboratory using *Prevotella heparinolytica*, which facilitates the progression of MM in mice and increases the levels of interleukin 17 (IL-17), a cytokine associated with a faster progression of disease (14, 29). In this sense, butyrate, a short chain fatty acid (SCFA) produced by gut microbiota, could play a major role since it can increase Tregs and suppress IL-17A. Interestingly, the butyrate producers *Eubacterium hallii* or *Faecalibacterium prausnitzii* have been associated to increased rates of minimal residual disease negativity (30). Nevertheless, the role of SCFAs in disease progression and response to treatment should be further explored, since the SCFA producers *Anaerostipes hadrus, Clostridium butyricum*, and *Clostridium saccharobutylicum* were reported as decreased in MM (16), while the butyrate producers *Clostridium leptum* and *Rothia* were increased (15). These microorganisms are the main bacteria involved in the glucose metabolism pathway, suggesting its dysregulation in MM patients (15). On the other hand, L-glutamine metabolism, and urea regulation by nitrogen-recycling bacteria have been recently proposed as a mechanism of MM progression (16).

Both reviews also focus on the role of gut microbiota in therapy response. Gut microbiota seems to enhance response to hematopoietic stem cell transplantation (HSCT), and could potentiate CAR-T therapy, checkpoint inhibitors, cyclophosphamide and bortezomib (31, 32). Thus, gut microbiota could reduce gastrointestinal toxicity associated with bortezomib (Brevi et al.) but also influence the risk of adverse events by modulating the NF-kB pathway (Jasiński et al.).

## Concluding Remarks

**Figure 1:**
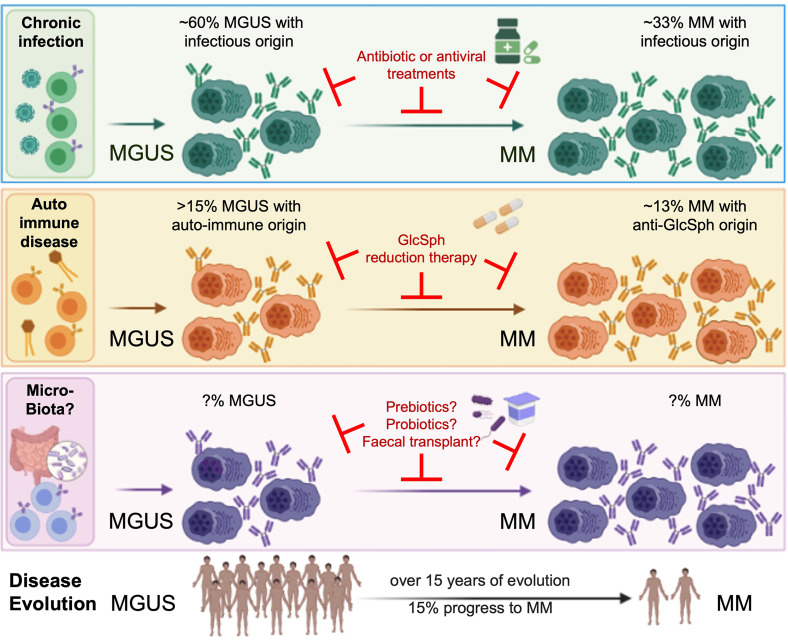
Micro-organisms as initiating events in the pathogenesis of MGUS and MM, and interest for treatment. MM is always preceded by a MGUS stage, considered benign since only a fraction of MGUS eventually progress toward to overt MM. Although MGUS is most often asymptomatic, patients may present with renal, neurological or skin symptoms of various severity (for instance, monoclonal gammopathies of renal significance (MGRS)) (6–8). Associations between MGUS and autoimmune disorders, infection and recently, gut microbiota, are established (3, 5, 9, 10, 14–22). Importantly, several groups demonstrated that monoclonal Ig from MGUS and MM patients specifically target either a self-antigen (including myelin-associated glycoprotein (MAG), glucosylsphingosine (GlcSph, in 13-15% MGUS/MM cases), other gangliosides, different membrane components) or an antigen from an infectious pathogen (including HCV, HBV, HIV, EBV, other Herpesviruses, *Helicobacter pylori*, or Enteroviruses, in 60% MGUS and 33% MM). That subsets of monoclonal Igs target antigens of the microbiota remains to be proven. Knowing the target of a patient’s monoclonal Ig is essential, for it allows to identify the likely initiating event of the disease, and to propose target antigen reduction therapy to patients. The efficacy of this new therapeutic approach is proven for GlcSph-, HIV- and HCV-initiated monoclonal gammopathies (22, 33, Rodríguez–García et al.), and deserves to be tested for MGUS and MM initiated by other treatable microorganisms.

Present evidence indicates that a prior history of autoimmune disease or chronic infection with certain pathogens increases the risk of infection-initiated MGUS and MM as represented in **Figure 1**. These findings will have major impact on the treatment of monoclonal gammopathies, since both MGUS and MM patients can benefit from antigen target reduction therapy, as demonstrated with antiviral treatment for HCV-initiated MGUS and MM.

On the other hand, a better understanding of the role played by gut microbiota in MM pathogenesis, disease progression and response to therapy is needed. It would allow to treat patients with prebiotics, probiotics or fecal microbiota transplantation (FMT), to reduce the risk of progression (**Figure 1**). In this context, a randomized trial was recently launched to assess FMT efficacy in preventing allogeneic-HSCT complications in MM patients (NCT04935684). 

## Author Contributions

The authors wrote the editorial and approved it for publication.

## Conflict of Interest

The authors declare that the research was conducted in the absence of any commercial or financial relationships that could be construed as a potential conflict of interest.

## Publisher’s Note

All claims expressed in this article are solely those of the authors and do not necessarily represent those of their affiliated organizations, or those of the publisher, the editors and the reviewers. Any product that may be evaluated in this article, or claim that may be made by its manufacturer, is not guaranteed or endorsed by the publisher.
